# Bispecific Chimeric Antigen Receptor T Cell Therapy for B Cell Malignancies and Multiple Myeloma

**DOI:** 10.3390/cancers12092523

**Published:** 2020-09-05

**Authors:** Robert J. Cronk, Joanna Zurko, Nirav N. Shah

**Affiliations:** Division of Hematology & Oncology, Medical College of Wisconsin, Milwaukee, WI 53005, USA; rcronk@mcw.edu (R.J.C.); jzurko@mcw.edu (J.Z.)

**Keywords:** CAR T cell, bispecific, NHL, ALL, myeloma, CD19, CD20, CD22, BCMA, CD38

## Abstract

**Simple Summary:**

In relapsed, refractory B cell malignancies and multiple myeloma, chimeric antigen receptor (CAR) T cell therapy has represented a major scientific advancement with high response rates and durable responses for many. Nonetheless, target antigen downregulation in tumor cells can lead to poor responses and relapsed disease. Current FDA approved CAR T cell therapies only target a single B-cell specific cell marker. While effective, single targeted CAR T cell therapy can lead selective pressure against the target antigen leading to loss of expression and tumor cell escape. Simultaneous dual antigen targeting CAR therapy has been evaluated in multiple early phase clinical trials in response to these clinical challenges in hopes of improving response rates and preventing relapse. This article discusses the limitations of single targeted CAR T cell therapy, approaches to dual antigen targeting, and the results of early phase trials utilizing dual antigen targeting CAR T cell therapy.

**Abstract:**

Chimeric antigen receptor (CAR) modified T cell therapy offers a targeted immunotherapeutic approach to patients with refractory hematological malignancies. This technology is most advanced in B cell malignancies and multiple myeloma and is rapidly evolving as more data become available regarding clinical efficacy and response durability. Despite excellent initial response rates with single antigen targeting CARs, failure to respond to therapy and relapse due to target antigen downregulation remain clinical challenges. To mitigate immunophenotypic selective pressures, simultaneous dual antigen targeting with bispecific CAR T cells or multiple administration of different populations of CAR T cells may prevent relapse by addressing one resistance mechanism attributed to antigenic loss. This article will review recently published data on the use of dual targeting with CAR T cells from early phase clinical trials aimed at treating B cell malignancies and multiple myeloma.

## 1. Introduction

In an era of rapidly expanding adaptive cellular immuno-therapies, chimeric antigen receptor (CAR) T cells have shown unprecedented results in the treatment of relapsed, refractory (R/R) hematologic malignancies. Through ex vivo modifications, autologous T cells are collected via leukapheresis, activated and expanded after being exposed to a viral vector that encodes for costimulatory domains attached to a monoclonal antibody-derived single-chain variable fragment (scFv) capable of targeting specific tumor-associated antigens [1,2]. Following lymphodepletion, these genetically modified CAR T cells are reinfused into the patient through autologous adoptive T cell transfer, exerting their effects independently of human leukocyte antigen (HLA) signaling. These modified T cells then target the specified tumor antigen and eliminate malignancy even in refractory cases. While effective, CAR T cell therapy can be complicated by a cytokine mediated inflammatory storm that can present with high fevers, confusion, hypotension, confusion, seizures, and in rare cases death. Management of these toxicities is aimed at blocking the cytokine cascade with tocilizumab, an anti-IL6 receptor blockade, the current standard of care [1].

### 1.1. First, Second and Third Generation CAR T Cell Products

As early prototypes consisting simply of the fusion product of a monoclonal antibody linked to the transmembrane and intracellular domains of the T cell receptor (i.e., CD3ζ), first generation CAR T cells lacked significant clinical efficacy and persistence in vivo [3]. Second generation CAR T products were able to enhance CAR T cell function by incorporating specific intracellular co-stimulatory domains—such as 4-1BB or OX40 of the tumor necrosis factor (TNF) receptor superfamily, or CD28 of the immunoglobulin (Ig) superfamily [4,5]. These products comprise much of the commercially available CAR T products on the market today. Limited data exist evaluating the effect of third generation CAR T products with incorporation of multiple co-stimulatory domains [6,7], though early phase data in CD19 targeting for B cell malignancies show excellent safety data and persistence of the CAR T cells beyond three months following infusion [8].

### 1.2. B-Cell Specific Cell Surface Marker Targets for CAR T Cells

Several B-cell-specific cell surface markers, CD19, CD20, and CD22 represent attractive targets for the development of adaptive cellular immunotherapies in the treatment of B cell malignancies. With FDA approval of multiple commercial CAR T products (tisagenlecleucel T, axicabtagene ciloleucel, brexucabtagene autoleucel), CD19 targeting remains the best characterized approach with impressive responses in heavily pre-treated populations [9,10,11,12]. Despite promising initial clinical efficacy, durability of response and non-response are ongoing clinical challenges. Adults with aggressive non-Hodgkin lymphoma (NHL) have only a 30–40% long-term progression free survival (PFS) after single targeted CD19 CAR T cell therapies [13]. In pediatric and young adult acute lymphoblastic leukemia (ALL), CD19 CAR T-cells manufactured with a lentiviral vector with 4-1BB and CD3ζ costimulatory domains, the 1-year event free survival (EFS) was only 50% [12]. Similarly, with regard to long-term follow-up in adult B cell ALL utilizing a CD19 CAR T cell product with CD28 and CD3ζ costimulatory domains, while the complete response (CR) was 83%, the median EFS was only 6.1 months with a median overall survival (OS) of 12.9 months [14]. Although CD19 CAR T cell therapy is now a standard approach in the management of R/R B-cell malignancies the above results demonstrate that the treatment is not a panacea.

For patients with multiple myeloma, the most common target under clinical investigation is the B-cell maturation antigen (BCMA). As BCMA expression is limited to the surface of plasma cells and plasmablasts, it serves as an ideal target for CAR T cell therapy with limited off target toxicities [15]. Early phase clinical trials with anti-BCMA CAR T cells have demonstrated overall response rates (ORR) spanning 20–100% with CR rates ranging from 11–76% [16,17].

### 1.3. Target Antigen Loss in CAR T Cell Therapy

Despite the exciting results seen with CAR T cell therapy in both B-cell malignancies and multiple myeloma, a major limitation of single targeted CAR T cell therapies is selective pressure against the target antigen leading to loss of expression and tumor cell escape [18]. This was first appreciated in patients with B cell ALL where CD19 negative relapse was observed as a resistance mechanism to CAR T cell therapy [12]. Studies have now shown that up to 25% of B cell ALL patients who initially respond to CD19 CAR therapy can relapse with a CD19 negative B cell clone [12,19,20,21,22,23]. In the NHL setting, CD19 expression data post-CAR T cell therapy are limited due to the need for tissue biopsy at the time of relapse and use of lower sensitivity methods of detection (i.e., immunohistochemistry, IHC) compared to flow cytometry (FC) that is typically used in B cell ALL [21]. Combining limited available data in the NHL setting, approximately a third of relapses exhibited CD19 loss on tissue biopsy [10,11,24,25]. The mechanism of antigenic loss is complex and variable depending on the patient. Several mechanisms have been proposed including acquired mutations in the CD19 gene leading to either no cell surface CD19 expression or a truncated CD19 protein that no longer contains the epitope targeted by CAR T-cells [26]. Alternatively, cell lineage switch to a phenotype that does not express the targeted antigen has been described as an alternative method to evade CAR T-cells. This finding has been best described in patients with mixed lineage leukemia rearranged B-cell ALL that relapses with CD19 negative acute myeloid leukemia after administration of CD19 CAR T-cell therapy [27]. Antigen downregulation is not limited to the CD19 receptor on B-cells. Similar to the finding of CD19 loss after CAR administration, early studies of CD22 targeted CARs demonstrated similar antigen escape in a phase 1 trial with patients with R/R B-cell ALL. Among eight patients who initially responded and subsequently relapsed, CD22 expression was diminished or absent in seven of the patients [28]. Lastly, sequential administration of single targeted CARs may result in similar antigenic loss. In a case of diffuse large B-cell lymphoma (DLBCL) treated initially with CD19 targeting CAR T cells, a patient developed CD19 negative relapsed disease. This patient was subsequently treated with CD22 targeted CAR T cells, which then resulted in antigenic loss of CD22, demonstrating the sequential loss of B cell antigens after CAR therapy and a potential limitation of a consecutive rather concurrent or dual targeting CAR approach [29]. Lastly, antigenic loss to evade CAR T cell therapies is not unique to B-cell malignancies with downregulation of BCMA being reported in early phase trials with BCMA directed CAR T cell therapy for multiple myeloma [30].

As target antigen loss or downregulation following CAR T cell treatment for R/R B cell malignancies and multiple myeloma is an established method of tumor resistance, targeting multiple antigens simultaneously represents a promising approach to help enhance efficacy and maximize response durability [21,31,32]. Preclinical data in B-cell malignancies have demonstrated that targeting more than one B-cell antigen (e.g., bispecific CARs) may not only decrease the risk of antigen escape of the targeted antigens but also non-targeted B-cell antigens and potentially improve response rates and eliminate one resistance mechanism [31,33]. These preclinical data have driven a significant expansion of CAR T cell trials that target more than one antigen. In this article, we will highlight data from early phase clinical trials of dual-targeted CAR T cells as a potential platform to mitigate target antigen downregulation and improve both response rates and durability of response. 

## 2. Approaches to Dual Antigen Targeting

**Figure 1:**
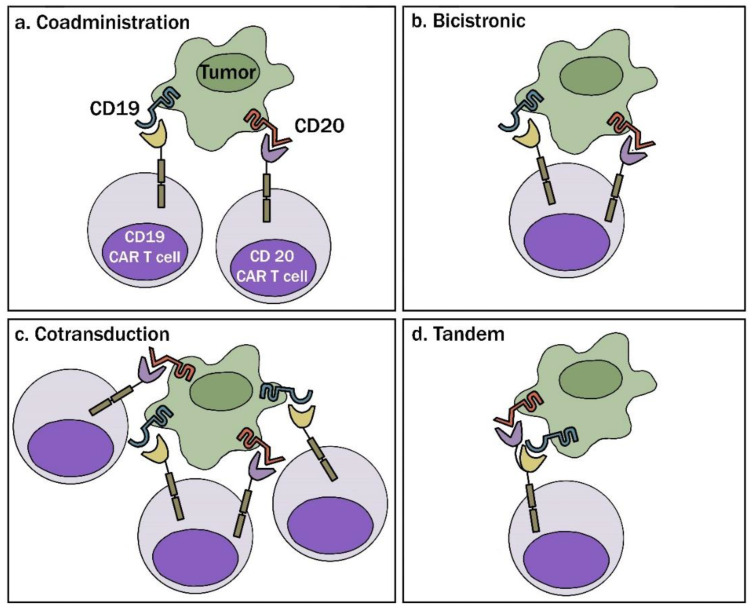
Dual antigen chimeric antigen receptor CAR T approaches. (**a**) Coadministration: involves production of two separate CAR T cell products infused together or sequentially. (**b**) Bicistronic: allows expression of two different CARs on the same cell. (**c**) Cotransduction: encodes two CAR constructs with multiple vectors. With this process, one will also obtain cells that express each CAR alone. (**d**) Tandem: encodes two CARs on same chimeric protein using a single vector. This work is licensed under a CC-BY Creative Commons attribution license, version 4.0 [32].

Dual targeting has been proposed as a mechanism to overcome target antigen loss as a mechanism of treatment failure with CAR T cell therapy. However, there are several strategies that can be utilized to target multiple antigens via CAR T cell therapy (Figure 1) [21,32]. First, either sequential or simultaneous coadministration of separately engineered T cell populations with unique CARs can be considered. Challenges to this approach include the need for >1 manufacturing run to generate individual CAR populations which can be both an inefficient and costly process. Co-transduction of two separate CAR vectors incorporated simultaneously yields a heterogenous product with up to three unique populations, including two separate subsets harboring one of each individual CAR construct, in addition to a third subset harboring both CARs. Tandem or bivalent CAR constructs can also be considered, which incorporate two distinct antigen-binding sites on a single extracellular domain. Lastly, bicistronic products are engineered using a single vector that encodes distinct, unique CARs to allow dual targeting through separate extracellular motifs [21,32]. At this time, the optimal method for dual targeting is unclear with all the aforementioned approaches under clinical investigation.

## 3. Dual Targeting in B-Cell Malignancies

### 3.1. Combined CD19 CD20 CARs

Given the clinical efficacy of CAR T cell therapy with CD19 targeting, most combinatorial studies have included CD19 targeting with additional B-cell antigens. CD20, a well-known B cell antigen, has been the target for monoclonal antibodies for decades and is felt to be an integral part of treatment for mature B-cell malignancies [34]. As a result, a natural combination and an area under active investigation in B cell NHL are bispecific constructs targeting both CD19 and CD20 [35]. Early results of a bispecific, tandem, anti-CD19 anti-CD20 lentiviral 4-1BB/CD3ζ CAR construct were recently reported. Using the CliniMACS Prodigy system for manufacturing, this bispecific tandem CAR was tested in a phase 1, dose escalation and expansion study in R/R B-cell NHL. The ORR was 82% with 55% achieving a CR and 27% achieving a PR at day 28 evaluation. Among the patients with relapsed or progressive disease, downregulation of targeted receptors was not observed, suggesting antigenic loss was not the etiology of treatment failure. The toxicity profile was promising with no dose-limiting toxicities (DLTs) among the 11 patients treated and no grade three quarter cytokine release syndrome (CRS) or neurotoxicity [35]. A recent publication reported results from a different tandem CD19-CD20 CAR T-cell in patients R/R B-cell NHL. Among the 28 patients who received a CAR T-cell infusion, the ORR was 79% with CR rate of 71%. The median PFS was not reached for treated patients and the PFS at 1 year was 64%. Grade 3 CRS occurred in 14% of patients and 17% required tocilizumab for management. There were no three quarter grade neurological toxicities [36]. An update of this study was provided in abstract form in June 2020 which reported on outcomes of 87 patients. Among this larger population while the efficacy signal remained stable, there were more reported toxicities with three treatment related deaths [37]. Several other trials utilizing this combination are actively accruing patients (NCT04007029 and NCT04186520).

### 3.2. Combined CD19 CD22 CARs

An alternative approach to CD20 targeting is combinatorial CD19-CD22 CAR T-cells. Similar to CD20, CD22 is expressed on most B-cell leukemias and lymphomas making it an attractive target for cellular immunotherapeutic treatments [38]. In a phase 1 study, a bicistronic construct linking a CD19 CAR to an OX40 costimulatory domain alongside a CD22 CAR linked to a 4-1BB costimulatory domain (AUTO3) was utilized for relapsed, refractory pediatric B-cell ALL [39]. Among seven evaluable patients, the CR rate was 100%, however, with a median of 8 months of follow-up, emergence of minimal residual disease by PCR was identified in four patients with one relapse noted to have CD19 loss and low CD22 expression 1 year out from treatment. Though there were no grade 3-4 CRS or neurotoxicity noted, 80% experienced a grade 1 CRS with 10% having grade 2 CRS. This same product, AUTO3, was also tested in patients in patients with R/R DLBCL in combination with pembrolizumab, an immune checkpoint inhibitor (ICI). Among 11 patients treated at a dose level >50 × 10^6^ cells/kg the ORR and CR rates were 64% and 55%, respectively. There were no cases of severe CRS or any neurotoxicity in this patient population [40].

Schultz et al. [41] presented their outcomes utilizing a bispecific, tandem anti-CD19, anti-CD22 4-1BB, CD3ζ CAR construct. In a phase 1 study for patients with R/R B-cell ALL, among 12 evaluable patients, 11 achieved a CR with one patient with primary progressive disease. Thus far three patients have relapsed, but all retained CD19 expression suggesting target antigen loss was not the primary mechanism of relapse. The toxicity profile of this product was favorable with only one patient with grade 4 CRS and neurotoxicity that resolved with management. Duration of response after CAR therapy is difficult to measure as six pediatric patients proceeded with a consolidative allogeneic transplant beyond day 28 [41]. Dai et al. [42] recently published their case series of six patients treated with a separate but similar tandem, bispecific CD19-CD22 CAR T-cell product in R/R B cell ALL. Minimal Residual Disease (MRD) negative remission was achieved in all six patients, however, three patients relapsed, one with CD19 negative and CD22 diminished disease which is concerning in the fact that that target downregulation can remain a problem even with a dual targeted construct. Toxicity was minimal with only grade 1–2 CRS among treated patients [42]. Lastly, Gardner et al. [43] reported outcomes utilizing a co-transduction approach to dual targeting of CD19 and CD22 for relapsed B-cell ALL. The manufactured product demonstrated that 22–26% of T-cells were positive for CD19 CAR, 31–39% had only CD22 CAR while 40–44% had both CD19 and CD20 CARs present. Interestingly after infusion of this product, there was preferential expansion of the CD19 CAR product over the single CD22 CAR or the CARs containing both CD19 and CD22 CARs. Five out of seven patients achieved a complete remission by day 21 and toxicity was mild with no grade 3–4 CRS or neurotoxicity [43].

As an alternative to the tandem, costransduction, and bicistronic products reviewed thus far, Pan et al. [44] evaluated sequential CD19 and CD22 CAR T cell infusions in a phase 1 trial involving 20 pediatric patients with R/R B-ALL [44]. In this study, CD19 targeting CAR T cells were infused initially. Once the CD19 product was no longer detectable in the peripheral blood, CD22 targeted CAR T cells were infused at a median of a 1.65-month interval between infusions. At day 30 after the CD19 CAR infusion, 100% of patients achieved an MRD negative CR that had persisted through infusion of the CD22-targeted product. No patient underwent consolidative allogeneic transplant. At the study endpoint, three patients (15%) relapsed, with antigenic loss of CD19 seen in two patients and CD22 downregulation seen in another. While target antigen loss still occurred, the overall clinical outcomes in this study compare favorably to historical results with single targeted CD19 CAR T cell therapy although limited by follow-up time. In terms of toxicity, grade ≥3 CRS and neurotoxicity were only seen after administration of the CD19 CAR T-cell product with only grade 1–2 CRS and neurotoxicity with the CD22 CAR T-cell product [44]. Table 1 summarizes bispecific CAR approaches in B-cell malignancies.

## 4. Dual Targeting in Multiple Myeloma

### 4.1. Combined CD19 BCMA CARs

Similar to B-cell malignancies, dual targeting approaches are being investigated in multiple myeloma. As distinct populations of myeloma cells have CD19 expression [50], and early reports have shown responses to CD19 targeted CAR T cell therapy with improved PFS in R/R multiple myeloma [51], initial dual targeted approaches focused on combinatorial CD19 and BCMA CARs as a potential strategy improve response and durability. Zhang et al. [46] utilized a bispecific tandem CAR T construct linking BCMA and CD19 for treatment of five adults with R/R multiple myeloma. While follow-up time is limited, all patients responded including stringent CR in one patient, very good partial response in three, and PR in one additional patient. Treatment was well tolerated with only grade 1 CRS reported in three patients with no neurotoxicity reported of any grade [46]. Another group recently published their phase 2 data describing simultaneous co-administration of humanized anti-CD19 CAR T cells along with murine anti-BCMA CAR T cells for treatment of R/R multiple myeloma. Among 21 treated patients, the ORR was 95% with 12 patients achieving a stringent CR or CR after infusion. Of these responding patients, most (85%) did not relapse at the time of publication. A total of 86% of patients developed grade 1–2 CRS with grade 3 CRS in 5%, along with 10% developing CAR-related encephalopathy syndrome [47]. While there was not a comparator arm, the high overall responses and durability of response is suggestive of a potential impact of combination therapy.

### 4.2. Combined CD38 BCMA CARs

As an antigen highly expressed on multiple myeloma cells, CD38 serves as a target for commercially available monoclonal antibodies that can be used in either the first-line setting or in the R/R setting with well-documented clinical efficacy [52,53]. Consequently, CAR constructs targeting CD38 are now under development. One group tested a tandem bispecific CAR T therapy targeting both BCMA and CD38 linked to 4-1BB and CD3ζ costimulatory domains as part of a phase 1 trial to assess efficacy, durability, and safety profiles in the R/R multiple myeloma setting. This dose-escalation study included 16 patients who received at least two prior lines of treatment for study enrollment. Overall response was 87.5%, with eight patients achieving a stringent CR. Nine-month PFS was 75%, with one patient maintaining stringent CR for longer than 51 weeks. There were no reports of neurotoxicity, although four patients required tocilizumab for management of grade 3–4 CRS with resolution. Grade 1–2 CRS occurred in 10 patients [48]. This study highlights the feasibility and promising efficacy of dual BCMA and CD38 targeting while maintaining an acceptable and manageable toxicity profile.

### 4.3. Combined BCMA-TACI CAR

Similar to BCMA, transmembrane activator and calcium-modulator and cyclophilin ligand interactor (TACI) is expressed on multiple myeloma cells. AUTO2 is a CAR T-cell designed to target both BCMA and TACI concurrently with a novel CAR construct using a truncated form of a proliferation-inducing ligand (APRIL) as the tumor targeting domain. In this phase 1 clinical trial, patients with R/R multiple myeloma were enrolled in a dose escalation trial. A total of 11 patients had achieved the minimum follow-up time and were evaluable for safety endpoint. A total of five patients had grade 1 CRS and there were no cases of neurotoxicity. Among patients who received ≥225 × 10^6^ cells dose, the ORR was 43% in this early phase trial [49]. Table 1 summarizes dual targeted CAR approaches in multiple myeloma.

## 5. Future Multi-Targeted CAR T Approaches

Despite the numerous approaches described above which are encouraging, the findings are all limited by low sample size and limited follow-up. Larger studies and ideally those comparing bispecific CARs directly to single targeted CARs are necessary to determine if dual targeting can improve the current standard of care for B-cell malignancies and multiple myeloma. Efforts for advancing the field of multi-targeting CAR T cell therapy press on in various directions, with CAR T therapies comprising over half of all hematologic malignancy trials with over a thousand cellular therapies currently under investigation [54].

## 6. Autologous vs Allogeneic Bispecific Approaches

Though multiple clinical trials are assessing efficacy of CD19-directed allogeneic CAR T cells in B-cell malignancies (NCT02808442, NCT02746952), there is a paucity of data regarding allogeneic approaches. However, there are substantial intrinsic advantage of allogeneic products. Specifically, these products can be available on demand and would not be dependent on patient apheresis for manufacturing which is a potentially lengthy process without a guarantee of successful CAR T cell production at the end. There is a phase 1 study patients in R/R B-cell malignancies that seeks to assess efficacy, safety, and feasibility of allogeneic stem cell transplant following allogeneic bispecific CD19/CD22 CAR T cells or single-targeted CD19 CAR T cells (NCT03463928). It is likely that once the role of single a targeted allogeneic CAR is established that similarly dual targeting will be studied. Time will tell if allogeneic products will be an alternative to autologous approaches or potentially replace autologous all together.

## 7. B-Cell Malignancies

Multiple alternative B-cell associated antigens are found to persist in CD19-negative relapse in B-cell malignancies, posing therapeutic opportunities that investigators hope to exploit in ongoing clinical trials. Additional antigenic targets under investigation include CD37 (4-passage transmembrane protein), CD10 (common acute lymphocytic leukemia antigen), TSLPR (thymic stromal lymphopoietin receptor), CD70 (protein expressed on highly-active B- and T-lymphocytes), and CD30 (TNF receptor-related surface protein expressed on activated B- and T-lymphocytes) [55,56,57,58,59]. Scarfo et al. [56] recently published their preclinical development of anti-CD37 single targeted CAR and anti-CD19/anti-CD37 dual targeted CAR T cells for B-cell malignancies. Two tandem constructs with 4-1BB and CD3ζ with a different order of anti-CD37 and anti-CD19 were tested. These studies demonstrated improved transduction efficiency with the CAR37-19 as compared to the CAR19-37. While both CARs were activated by CD19 alone or CD37 alone, there was complete eradication of tumor in mice models with the CAR37-19 while only a partial clearing with the CAR19-37 construct [56].

In an early phase 1 study, one group in Guangdong, China plans to assess sequential treatment with either CD20-, CD22- or CD10-CAR T cells following CD19-CAR T therapy in 30 patients in the R/R B-ALL setting (NCT03407859). Another group is working to assess multiple non-CD19 targeting CAR T products in a combined phase 1/2 study in 100 patients with CD19-negative B-cell malignancies (NCT04016129). Patients with either de novo CD19-negative disease or CD19-negative relapse after CD19-CAR T therapy that express one or more antigens of interest (CD22, CD123, CD38, CD10, CD20 and/or TSLPR) will receive either one or multiple non-CD19 targeting CAR T products. Results from these studies will be informative on the impact of dual or multi-targeting products on clinical outcomes.

## 8. Multiple Myeloma

In parallel to ongoing efforts in B-cell malignancies, numerous plasma cell-associated antigens are under investigation for use in bispecific CAR T therapy for the treatment of R/R multiple myeloma. Several promising novel targets have emerged, including CD138 (adhesion protein binding collagen and fibronectin in the extracellular matrix, known as syndecan 1), integrin β7 (principal regulator in cell-extracellular matrix and cell-cell interactions), CS1 (plasma cell marker encoded by SLAMF7 gene), and CD56 (neural cell adhesion molecule) [60]. Preclinical data for a dual targeted CAR against BCMA and CS1 demonstrated individual activity against cell lines that were BCMA+CS1- and BCMA-CS1+. In-vivo data revealed sustained activity and superior murine survival when compared to single BCMA targeting CAR T-cell therapy [61]. To further enhance the activity of a dual targeted BCMA-CS1 CARs, administration of concurrent anti-PD1 has been tested preclinically resulting in augmented speed of in-vivo tumor clearance [62]. Phase 1 studies are currently enrolling patients with multiple myeloma to evaluate the safety and efficacy of either single CAR T cell products targeting BCMA, CD138, CD38, Integrin β7 or CS1, or ten different dual-targeting combinations of these products (NCT03778346). Additionally, multiple groups are currently studying various combinations of either single- or multi-targeted CAR T cells with antigens of interest including CD138, BCMA, CD19, CD38, CD56 or other unspecified antigens (NCT03196414, NCT03271632, NCT03473496).

## 9. Tri-Specific CAR T Cells

Building on the successes of dual antigen targeting, tri-specific CAR T cells are under evaluation for the treatment of hematologic malignancies [33]. Through tri-specific targeting of CD19, CD20 and CD22, Fousek et al. [33] developed two separate constructs; one with three distinct monovalent second generation CARs (TriCAR), and the second with a monovalent CD19-targeting CAR and an additional bivalent CAR with scFvs dually targeting CD20 and CD22 (SideCAR). Pre-clinical experimentation with both tri-specific products suggests levels of secretion of IFN-γ and TNF-α are similar to that of single-targeting CD19 CAR T cells, but with the tri-specific products demonstrating more robust malignant cell killing than what is seen with the monovalent comparator. The tri-specific products were also tested in models of CD19-negative relapse and CRISPR-mediated CD19 knockouts, demonstrating effective cytokine production and enhanced killing of CD19-negative ALL cells, suggesting lessened incidence of antigenic-escape [33].

## 10. Conclusions

CAR T cell therapy has reshaped treatment paradigms for both B cell malignancies and multiple myeloma in the R/R setting. Despite excellent initial clinical efficacy, malignant cells exercise a myriad of resistance mechanisms to evade CAR T cell directed cytotoxicity. Most commonly, antigenic loss and target receptor downregulation have been reported after administration of single targeting CAR T cell products. Dual targeting of more than one tumor antigen is now being studied in clinical trials to improve efficacy of treatment and mitigate antigen loss. The ideal combinations of antigens to target or if multi-targeting improves clinical outcomes remains an unanswered question. Preliminary data from early phase studies with dual targeting demonstrate excellent response rates with manageable toxicities, albeit with short follow up intervals. Longer follow-up with a greater number of patients is indicated to determine whether bispecific approaches can reduce risk of relapse from antigen downregulation and improve outcomes when compared to currently available single targeted CAR therapies.

## Figures and Tables

**Table 1:** Bispecific CAR T cell therapies in relapsed/refractory B-cell malignancies and multiple myeloma.

Trial	Patient Population	Target/Construct	Lymphodepletion	Dose Range	Response	Follow Up	Toxicity	Treatment of Toxicity
**B-cell Malignancies**								
Shah et al. [35] Phase 1	Adult R/R NHL DLBCL (*n* = 5) MCL (*n* = 4) CLL (*n* = 2)	Tandem/bivalent CD19/CD20; 2nd generation with 4-1BB+ CD3ζ costim domains	Flu 30 mg/m^2^ × 3 d Cy 500 mg/m^2^ × 1 d	2.5 × 10^5^ to 2.5 × 10^6^ cells/kg	82% ORR ^1^ 55% CR 27% PR	Range 1–9 m	No DLTs 55% Gr 1–2 CRS 27% Gr 1–2 NTX	4 patients treated with toci for CRS
Zhang [37]	Adult R/R NHL (*n* = 74)	Tandem/bivalent CD19/CD20; 2nd generation with with 4-1BB and CD3ζ costim domains	Not specified	0.5 × 10^6^ to 10 × 10^6^ cells/kg	84% ORR 74% CR mPFS NR mOS NR	Median 13.5 m	61% Gr 1–2 CRS 10% Gr 3–4 CRS 2% Gr 3 CRES 4% TRD	Not specified
Osborne et al. [40]	Adult R/R DLBCL or transformed DLBCL (*n* = 18)	Bispecific/bicistronic CD19/CD22; 2^nd^ generation with costim domains of OX40 paired with CD19 and 4-1BB paired with CD22	Flu and Cy, doses not specified	50 × 10^6^ to 450 × 10^6^ cells	At > 50 × 10^6^ dose: ORR 64% CR 55% At 450 × 10^6^ dose: CR 67%	Not specified	No DLTs No severe CRS 5% severe NTX	Not specified
Amrolia et al. [39] Phase 1/2	Pediatric R/R B-ALL (*n* = 10)	Bispecific/bicistronic CD19/CD22; 2^nd^ generation with costim domains of OX40 paired with CD19 and 4-1BB paired with CD22	Flu 30 mg/m^2^ × 4 d Cy 500 mg/m^2^ × 2 d	1 × 10^6^ to 5 × 10^6^ cells/kg	100% CR/CRi 100% MRD- ^2^	Median 8 m, Range 2–12 m	No DLTs 80% Gr 1 CRS 10% Gr 2 CRS 10% Gr 1 NTX No Gr 3–4 CRS or NTX	1 patient treated with toci
Schultz et al. [41] Phase 1	Parallel Pediatric and Adult R/R B- ALL (*n* = 12)	Tandem/bivalent CD19/CD22; 2nd generation with 4-1BB costim domain	Flu and Cy, doses not specified	1 × 10^6^ to 3 × 10^6^ cells/kg	92% CR ^3^ 92% OS	Median 9.5m, Range 1–20 m	75% Gr 1–2 CRS 17% Gr 1–2 ICANS 8% Gr 4 CRS 8% Gr 4 ICANS	Not specified
Dai et al. [42] Phase 1	Adult R/R B-ALL (*n* = 6)	Tandem/bivalent CD19/CD22; 2nd generation with 4-1BB and CD3ζ costim domains	Flu 30 mg/m^2^ × 3 d Cy 30 mg/kg × 2 d	1.7 × 10^6^ to 3 × 10^6^ cells/kg	100% CR ^4^ (MRD-)	Range 3–11 m	67% Gr 1 CRS 33% Gr 2 CRS No NTX	2 patients treated with toci for Gr 1 CRS
Gardner et al. [43] Phase 1	Pediatric and young adult ALL (*n* = 7)	Cotransduction with CD19 and CD22 lentiviral vectors	Not specified	1.1 × 10^6^ to 3 × 10^6^ cells/kg	71% CR ^5^	Not specified	71% Grade 1 CRS 29% Grade 1 NTX	4 patients received toci +/− dexamethasone
Yang et al. [45] Phase 1	Adult R/R B-ALL (*n* = 11)	Bispecific CD19/CD22; 2nd generation with 4-1BB costim domain	Flu 30 mg/m^2^ × 3 d Cy 250 mg/m^2^ × 3 d	2.5 × 10^5^ to 5 × 10^6^ cells/kg	25% CR ^6^ (MRD+) in low-dose 100% CR ^6^ (86% MRD-) in med-dose High-dose endpoint not reached at time of analysis	Median 60 d, Range 7–139 d	75% Gr 1 CRS in low-dose cohort 71% Gr 1 CRS in med-dose cohort 14% Gr 2 CRS in med-dose cohort No Gr 3–4 CRS No NTX of any grade	Not specified
Pan et al. [44] Phase 1	Pediatric R/R B-ALL (*n* = 20)	Sequential/Co-administration CD19 followed by CD22	Not specified	10 × 10^5^ cells/kg	100% CR ^7^ (MRD-)	Not specified	15% Gr 1 NTX in both infusions CD19 CAR: 85% Gr 1–2 CRS 5% Gr ≥ 3 CRS 5% Gr 3 NTX CD22 CAR: 75% Gr 1–2 CRS	Not specified
**Multiple Myeloma**								
Zhang et al. [46] Phase 1	Adult R/R MM (*n* = 5)	Bispecific/Tandem BCMA/CD19; 2nd generation with CD3ζ costim domain	Flu and Cy × 3 d, doses not specified	1 × 10^6^ to 2 × 10^6^ cells/kg	20% sCR 60% VGPR 20% PR	Median 68 d, Range 27–144 d	No Gr 3–4 CRS reported 60% Gr 1 CRS No NTX reported	Supportive care
Yan et al. [47] Phase 2	Adult R/R MM (*n* = 21)	Simultaneous/Co-administration Humanized anti-CD19, Murine anti-BCMA	Flu 30 mg/m^2^ × 3 d Cy 750 mg/m^2^ ×1 d	1 × 10^6^ cells/kg	95% ORR ^8^ 43% sCR 14% CR 24% VGPR 14% PR 5% SD	Median 179 d, Range 17–602 d	86% Gr 1–2 CRS 5% Gr 3 CRS 10% NTX degree unspecified	1 patient received toci and 5 patients received steroids
Li et al. [48] Phase 1	Adult R/R MM (*n* = 16)	Bispecific/Tandem BCMA/CD38; 2nd generation with 4-1BB and CD3ζ costim domains	Flu 25 mg/m^2^ × 3 d Cy 250 mg/m^2^ × 3 d	0.5 × 10^6^ to 4 × 10^6^ cells/kg	50% sCR 12.5% VGPR 25% PR 87.5% had MRD- BM evaluations 9 m PFS 75%	Median 36wks	No DLTs 62.5% Gr 1–2 CRS 25% Gr 3–4 CRS	4 patients treated with toci and supportive care with resolution of CRS
Popat et al. Phase 1 [49]	Adult R/R MM (*n* = 7)	APRIL CAR Construct targeting BCMA and TACI with endodomains of CD28, OX40, and CD3ζ	Flu 30 mg/m^2^ × 3 d Cy 300 mg/m^2^ × 3 d	15 × 10^6^ to 900 × 10^6^ transduced CAR T cells	43% ORR, 27% PR and 14% VGPR		No DLTs, 5 patients with grade 1 CRS, no neurotoxicity	3 patients received toci

Legend: NHL = non-Hodgkin lymphoma; DLBCL = diffuse large B-cell lymphoma; ALL= acute lymphoblastic leukemia, MCL = mantle cell lymphoma; CLL = chronic lymphocytic leukemia; MM = multiple myeloma, R/R = relapsed/refractory; Flu = Fludarabine; Cy = Cyclophosphamide; ORR = overall response rate; CR = complete response; CRi = CR with incomplete hematologic recovery; sCR = stringent CR; VGPR = very good partial response; PR = partial response; MRD = minimal residual disease; BM = bone marrow; DLTs = dose-limiting toxicities; Gr = grade; CRS = cytokine release syndrome; NTX = neurotoxicity; ICANS = immune-effector cell neurotoxicity syndrome; Toci = Tocilizumab. ^1^ ORR (CR and PR) at day 28; ^2^ CR/CRi reported only in CAR naïve patients (9 of 10 patients) and only in those with minimum of 8 weeks of follow up (7 of 9 patients); ^3^ 10 of 12 patients with CR at day 28 and one patient with PR at day 28 which improved to CR by 180 without further intervention; ^4^ Rate of CR at day 30; ^5^ Rate of CR at day 21; ^6^ Rate of CR at day 15; ^7^ Rate of CR at day 30; ^8^ Responses reported as best response achieved at time of re-evaluation of activity (done at 2 weeks, 1 month, 2 months, 3 months, 6 months, and 1 year). Day 30 ORR was 95% with 19/20 with objective response (1 of 21 patients died of cerebral hemorrhage after day 14 PR).
